# Medical cannabis authorization patterns, safety, and associated effects in older adults

**DOI:** 10.1186/s42238-022-00158-5

**Published:** 2022-09-22

**Authors:** Laura MacNair, Maja Kalaba, Erica N. Peters, Matthew T. Feldner, Graham M. L. Eglit, Lucile Rapin, Cynthia El Hage, Erin Prosk, Mark A. Ware

**Affiliations:** 1 Canopy Growth Corporation, Toronto, Ontario Canada; 2 Santé Cannabis, Quebec, Canada

**Keywords:** Medical cannabis, Older adults, Safety, Pain

## Abstract

**Background:**

Use of medical cannabis is increasing among older adults. However, few investigations have examined cannabis use in this population.

**Methods:**

We assessed the authorization patterns, safety, and effects of medical cannabis in a sub-analysis of 201 older adults (aged ≥ 65 years) who completed a 3-month follow-up during this observational study of patients who were legally authorized a medical cannabis product (*N* = 67). Cannabis authorization patterns, adverse events (AEs), Edmonton Symptom Assessment Scale-revised (ESAS-r), and Brief Pain Inventory Short Form (BPI-SF) data were collected.

**Results:**

The most common symptoms for which medical cannabis was authorized were pain (159, 85.0%) and insomnia (9, 4.8%). At baseline and at the 3-month follow-up, cannabidiol (CBD)-dominant products were authorized most frequently (99, 54%), followed by balanced products (76, 42%), and then delta-9-tetrahydrocannabinol (THC)-dominant products (8, 4.4%). The most frequent AEs were dizziness (18.2%), nausea (9.1%), dry mouth (9.1%), and tinnitus (9.1%). Significant reductions in ESAS-r scores were observed over time in the domains of drowsiness (*p* = .013) and tiredness (*p* = .031), but not pain (*p* = .106) or well-being (*p* = .274). Significant reductions in BPI-SF scores over time were observed for worst pain (*p* = .010), average pain (*p* = .012), and overall pain severity (*p* = 0.009), but not pain right now (*p =* .052*)* or least pain (*p* = .141).

**Conclusions:**

Overall, results suggest medical cannabis was safe, well-tolerated, and associated with clinically meaningful reductions in pain in this sample of older adults. However, the potential bias introduced by the high subject attrition rate means that all findings should be interpreted cautiously and confirmed by more rigorous studies.

## Introduction

The use of medical cannabis is increasing for a variety of indications and symptoms, including chronic pain, anxiety, sleep, chemotherapy-induced nausea and vomiting, multiple sclerosis, epilepsy, and Parkinson’s disease (Hill 2019; Lim et al. 2017; Whiting et al. 2015). One of the fastest-growing segments of cannabis use is in the older adult population (aged 65 years and older), where cannabis use has increased 10-fold between 2012 and 2019 in Canada and 2-fold between 2015 and 2018 in the United States (notably, these increases occurred during a period of increased medical and recreational legalization) (Han and Palamar 2020; Statistics Canada 2019). Cross-sectional surveys suggest older adults generally used cannabis for pain and sleep disorders, use had a positive impact on their lives, and they were less likely to report problematic cannabis use than younger adults (Brown et al. 2020; Haug et al. 2017; Reynolds et al. 2018; Yang et al. 2021). Moreover, a systematic review and meta-analysis of cannabinoid-based medicines in older adults (≥ 50 years of age) concluded that they were safe and acceptable in this population (Velayudhan et al. 2021). However, only one longitudinal study has examined the safety and effectiveness of medical cannabis in this population, where patients over 65 years of age at a medical cannabis clinic in Israel were followed for 6 months and pain intensity, quality of life, and adverse events (AEs) were monitored (Abuhasira et al. 2018). Here, 93.7% of respondents reported an improvement in their condition and the most common AEs were dizziness and dry mouth.

The multiple therapeutic effects from these cannabis products are typically attributed to two cannabinoids: delta-9-tetrahydrocannabinol (THC) and cannabidiol (CBD). THC is a partial agonist at cannabinoid receptors 1 (CB1) and 2 (CB2) (Pertwee 2008), which influence multiple physiological processes, including pain, regulation of stress, inflammation, and sleep-wake cycles (Lutz et al. 2015; Maccarrone et al. 2015; Woodhams et al. 2015). CBD lacks appreciable functional activity at CB1 and CB2, but has > 60 molecular targets, which may contribute to its anxiolytic, anti-epileptic, analgesic, and anti-inflammatory effects (Ibeas Bih et al. 2015). Given the non-traditional access regimen for medical cannabis products, and the wide range of product formulations and potential therapeutic applications, real-world evidence (RWE) studies are critical to gain insight into the authorization patterns, safety, and associated effects of medical cannabis products. Although randomized controlled trials (RCTs) are the gold standard in demonstrating treatment efficacy, they are costly, and the results of RCTs are often difficult to generalize to a broad range of patients, providers, and healthcare settings. RWE studies may extend highly controlled RCTs via higher levels of external validity and generalizability (Gruden et al. 2016). RWE is therefore an important complement for the study of medical cannabis. While several observational studies of medical cannabis support its safety and effectiveness in specific therapeutic areas, few have examined the safety and effectiveness of medical cannabis in the older adult population, or as a function of its cannabinoid content (Booth and Tannock 2014; Bouso et al. 2020; Casarett et al. 2019; Kim et al. 2019; Li et al. 2019; Takakuwa et al. 2020). Here, we aimed to describe physician authorization patterns of medical cannabis products from Spectrum Therapeutics, a Canadian licensed producer of medical cannabis, and observe the safety and self-reported effectiveness of medical cannabis among older adults (≥ 65 years of age) from a network of medical cannabis clinics.

## Methods

### Study design and setting

The parent study was an observational study of patients who were screened, authorized for medical cannabis treatment, and followed at Santé Cannabis, a network of four medical cannabis clinics in Quebec, Canada (Kalaba et al. 2021). Patients were either referred to the clinic by their physician or self-referred. Study procedures were carried out by medical office associates, clinic coordinators, research assistants, physicians, and nurses during structured assessments and care planning during initial and follow-up visits at Santé Cannabis clinics (Prosk et al. 2021). Registered nurses conducted a review of the patient medical file, completed a medical history, measured vital signs, height, and weight (if on site), determined the primary symptoms for treatment, and assessed the appropriateness of cannabinoid-based medicine. At an initial clinic appointment, physicians confirmed the patient eligibility for medical cannabis. Currently, cannabis is not regulated as a medicine in Canada. Based on Health Canada guidelines for authorizing medical cannabis, two primary criteria are taken into consideration when assessing eligibility. First, the patient has already failed other treatment modalities. Second, the patient does not have any of the following contraindications: pregnant, breastfeeding, history of psychosis, diagnosis of schizophrenia, chronic obstructive pulmonary disease, unstable cardiovascular disease, or history of substance abuse or dependence. Next, physicians completed a medical cannabis authorization form that included the total recommended daily amount of cannabis in grams. Patients were recommended specific medical cannabis products (i.e., CBD-dominant, balanced [1:1 THC:CBD], and THC-dominant) and were provided with patient education (i.e., dosing and titration instructions). Numerous factors are considered when authorizing the daily amount of cannabis, product profile, and product format (e.g., medical assessment, age, occupational status, socio-economic status, potential drug-drug interactions). The product recommendations are based on a treatment protocol developed by the clinic stemming from previous clinical experiences, current scientific literature, and guidelines provided by both the College of Family Physicians of Canada (2021) and Health Canada (2018).

Follow-up visits occurred at 3, 6, 9, and 12 months after the initial appointment or on an as-needed basis. Follow-up visits were conducted by nurses and physicians to assess treatment adherence and effectiveness, AEs, changes in patient health status (primary and secondary symptoms), and educate patients on their treatment plan. During the course of treatment, adjustments related to dosing, frequency and type of cannabis product, and licensed producer may have been needed. All such changes were approved by the physician and were documented in the Electronic Medical Record patient file. In general, the first months of treatment constituted a titration period in which the treatment started at a low dose and was adjusted at every visit to monitor potential side effects and reach effective doses. This study was approved by the McGill University Institutional Review Board. A waiver of consent was required and approved by the ethics committee and by *La commission d'accès à l'information* of Quebec.

### Sample

In contrast to the previous report that focused on the entire sample, this analysis focused specifically on older adults (≥ 65 years old) who were authorized a medical cannabis product from Spectrum Therapeutics during an initial visit between October 2017 and August 2019. Demographics, history of cannabis use, and primary symptoms at baseline are shown in Table 1. The focus on older adults offers a unique extension to prior work. Specifically, our prior findings (i.e., that pain, tiredness, drowsiness, anxiety, and well-being improved over time and that medical cannabis was well tolerated among adults generally; Kalaba et al. 2021) cannot be assumed to generalize from the broader sample of the parent study to older adults specifically.

**Table 1:** Baseline characteristics of baseline only and complete cases

Characteristic	Overall (*N* = 187)^a^	Baseline only (*N* = 122)^a^	Complete (3 months) (*N* = 65)^a^	***p***-value^b^
**Age (years)**	73.38 (6.83)	74.00 (7.23)	72.22 (5.91)	0.072
**Sex**				0.38
Male	65 (35%)	45 (37%)	20 (31%)	
Female	121 (65%)	76 (63%)	45 (69%)	
**Occupational status**				0.38
Full time	9 (5.0%)	7 (5.8%)	2 (3.3%)	
Long-term disability	4 (2.2%)	2 (1.7%)	2 (3.3%)	
Other	1 (0.6%)	1 (0.8%)	0 (0%)	
Part time	10 (5.6%)	7 (5.8%)	3 (5.0%)	
Retired	151 (84%)	100 (83%)	51 (85%)	
Short-term diability	2 (1.1%)	0 (0%)	2 (3.3%)	
Unemployed	3 (1.7%)	3 (2.5%)	0 (0%)	
**History of dried cannabis use**				0.26
Current	4 (2.2%)	3 (2.5%)	1 (1.5%)	
Never	123 (66%)	85 (70%)	38 (58%)	
Occasional	4 (2.2%)	1 (0.8%)	3 (4.6%)	
Previous	50 (27%)	29 (24%)	21 (32%)	
Regularly	5 (2.7%)	3 (2.5%)	2 (3.1%)	
**Product profile**				0.35
CBD-dominant	99 (54%)	68 (58%)	31 (48%)	
Balanced	76 (42%)	46 (39%)	30 (46%)	
THC-dominant	8 (4.4%)	4 (3.4%)	4 (6.2%)	
**Primary symptom**				0.79
Fatigue	1 (0.5%)	1 (0.8%)	0 (0%)	
Insomnia	9 (4.8%)	6 (5.0%)	3 (4.6%)	
Mental health	3 (1.6%)	1 (0.8%)	2 (3.1%)	
Nausea/vomiting	2 (1.1%)	2 (1.7%)	0 (0%)	
Other	8 (4.3%)	5 (4.1%)	3 (4.6%)	
Pain	159 (85%)	104 (86%)	55 (85%)	
Seizures	1 (0.5%)	0 (0%)	1 (1.5%)	
Weight loss or lack of appetite	3 (1.6%)	2 (1.7%)	1 (1.5%)	
**BPI: worst pain**	8.00 (7.00, 9.00)	8.00 (7.00, 8.75)	8.00 (7.00, 9.00)	0.40
**BPI: least pain**	4.00 (2.00, 6.00)	4.00 (2.00, 6.00)	3.50 (2.25, 5.00)	0.95
**BPI: average pain**	7.00 (5.00, 8.00)	7.00 (5.00, 8.00)	6.88 (5.00, 7.00)	0.63
**BPI: pain right now**	5.00 (4.00, 7.00)	5.00 (4.00, 7.00)	5.00 (4.00, 8.00)	0.31
**BPI: pain severity**	5.81 (1.99)	5.74 (2.03)	5.93 (1.91)	0.57
**ESAS: pain**	5.00 (4.00, 7.00)	6.00 (3.00, 7.00)	5.00 (4.00, 7.00)	0.75
**ESAS: tiredness**	6.00 (4.00, 8.00)	5.00 (3.00, 7.00)	6.00 (4.00, 8.00)	0.14
**ESAS: drowsiness**	2.00 (0.00, 5.00)	2.00 (0.00, 5.00)	2.50 (0.00, 5.00)	0.83
**ESAS: well-being**	5.14 (2.63)	5.23 (2.80)	4.96 (2.27)	0.50
**THC dose (mg)**	2.00 (0.40, 6.00)	2.00 (0.40, 6.00)	4.00 (0.60, 6.00)	0.53
**CBD dose (mg)**	8.00 (6.00, 12.00)	8.00 (4.00, 12.00)	8.00 (6.00, 12.00)	0.40

^a^Mean (SD); *n* (%); median (IQR)

^b^Welch two-sample *t*-tests, Pearson’s chi-squared tests, Fisher’s exact tests, and Wilcoxon rank sum tests were used as appropriate

### Outcomes

Outcomes were assessed at baseline and at 3-, 6-, 9-, and 12-month follow-up in-person appointments. Outcome analyses were limited to baseline and 3-month follow-up data due to a high rate of attrition by month 6 (> 85% attrition). Physician authorization patterns were assessed in terms of dose, defined as the mean authorized daily dose (mgs) of THC and CBD. To assess safety, AEs were coded using the Medical Dictionary for Regulatory Activities (MedDRA) version 23 into System Organ Class (SOC) and Preferred Term (PT). To assess effects associated with use of medical cannabis, the Edmonton Symptom Assessment Scale-revised (ESAS-r) (Hui et al. 2015; Watanabe et al. 2011) and Brief Pain Inventory (BPI-SF) (Jumbo et al. 2021; Keller et al. 2004) were used. There was a low rate of endorsement of symptomatic levels on ESAS-r items measuring nausea, lack of appetite, shortness of breath, depression, and anxiety and so these items were not analyzed in this study.

### Statistical analysis

Mean and standard deviations are provided for continuous variables and percentages for categorical variables. All available data from the baseline and 3-month follow-up assessments were included in this analysis.

Mixed effects models with random intercepts for the participant and a dummy-coded predictor for time were fit to evaluate baseline and 3-month change in THC dose, CBD dose, ESAS-r items, and BPI-SF items. Data were included for these analyses if participants had complete data on medical cannabis use outcomes at baseline. CBD and THC dose variables were log transformed owing to positive skew and based on evaluation of model fit, heterogeneous residual variances were allowed for CBD dose. Regression coefficients predicting CBD and THC doses were subsequently back-transformed and expressed on the raw dose scale. Initial inspection of outcome variable distributions revealed substantial zero inflation (i.e., greater than expected frequency of zero values assuming a normal distribution) on ESAS drowsiness, reflecting non-symptomatic levels, likely due to the mixed clinical presentation of the sample. To account for this, zero-inflated Gaussian mixed effects models were fit for these outcomes, with random intercepts and fixed effects of time included on both zero-inflated and semi-continuous parts of the model. In addition, due to the presence of outliers on BPI worst pain, BPI average pain, and THC dose, robust mixed effects models using squared robustness weights with high robustness, but lower efficiency (*k* = 1.345) were fit.

As an exploratory analysis, we also evaluated differences across baseline product profile groups over time. Given the low prevalence of THC-dominant product use (*n* = 8, 4.4%), comparisons were limited to CBD-dominant vs. balanced product profiles. Main effects of and interactions with time for baseline product profile group were added to the models described above for ESAS-r and BPI-SF outcomes, with the CBD-dominant group serving as the reference category. Outcome difference due to product profile was measured as the difference in the change from baseline between CBD-dominant and Balanced product profile groups (i.e., the product profile × time interaction term). A positive interaction suggested a more favorable outcome for CBD-dominant relative to balanced product profile and a negative interaction a more favorable outcome for balanced vs. CBD-dominant product profile.

There was a high rate of attrition (65%) at month 3. To render the data more consistent with the missing at random assumption, multiple imputation was used, with an inclusive approach adopted for missing data models. Missing data models included baseline demographic characteristics (age, sex, occupational status), baseline cannabis use characteristics (history of dried cannabis use, product profile), and primary presenting symptom. To account for the possibility that some participants may have been lost to follow-up due to experiencing an AE and discontinuing medical cannabis, number of AEs was included as a missing data predictor as well. For inclusion of time-varying measures (i.e., CBD dose, THC dose, BPI-SF outcomes, and ESAS-r outcomes) in missing data models, the cross-lagged approach of Van Buuren (2018) was used to produce more tractable missing data models. Using this approach, all measures from the same time point were included as predictors of missing values of one another (e.g., BPI pain severity at month 0 and ESAS tiredness at month 0) in addition to all scores from the same measure at different time points (i.e., BPI pain severity at month 0 and BPI pain severity at month 3), assuming that the associations between cross-lagged predictors (e.g., BPI pain severity at month 0 and ESAS tiredness at month 3) would be captured through their non-cross-lagged counterparts. Passive imputation was used for the BPI pain severity outcome. Twenty iterations were used in the imputation process and 25 imputed datasets were generated. Analysis models from each imputed dataset were pooled following Rubin’s rules (Rubin, 2004).

All analyses were conducted in R version 4.12 (R Core Team 2021). The mice package (van Buuren and Groothuis-Oudshoorn 2011) was used for multiple imputation, the nlme package for mixed effects models (Pinheiro et al. 2021), glmmTMB package for zero-inflated mixed effects models (Brooks et al. 2017), the robustlmm package for robust mixed effects models (Koller 2016), and the emmeans package for estimated marginal mean calculation (Lenth et al. 2021).

## Results

### Sample characteristics

**Fig. 1:**
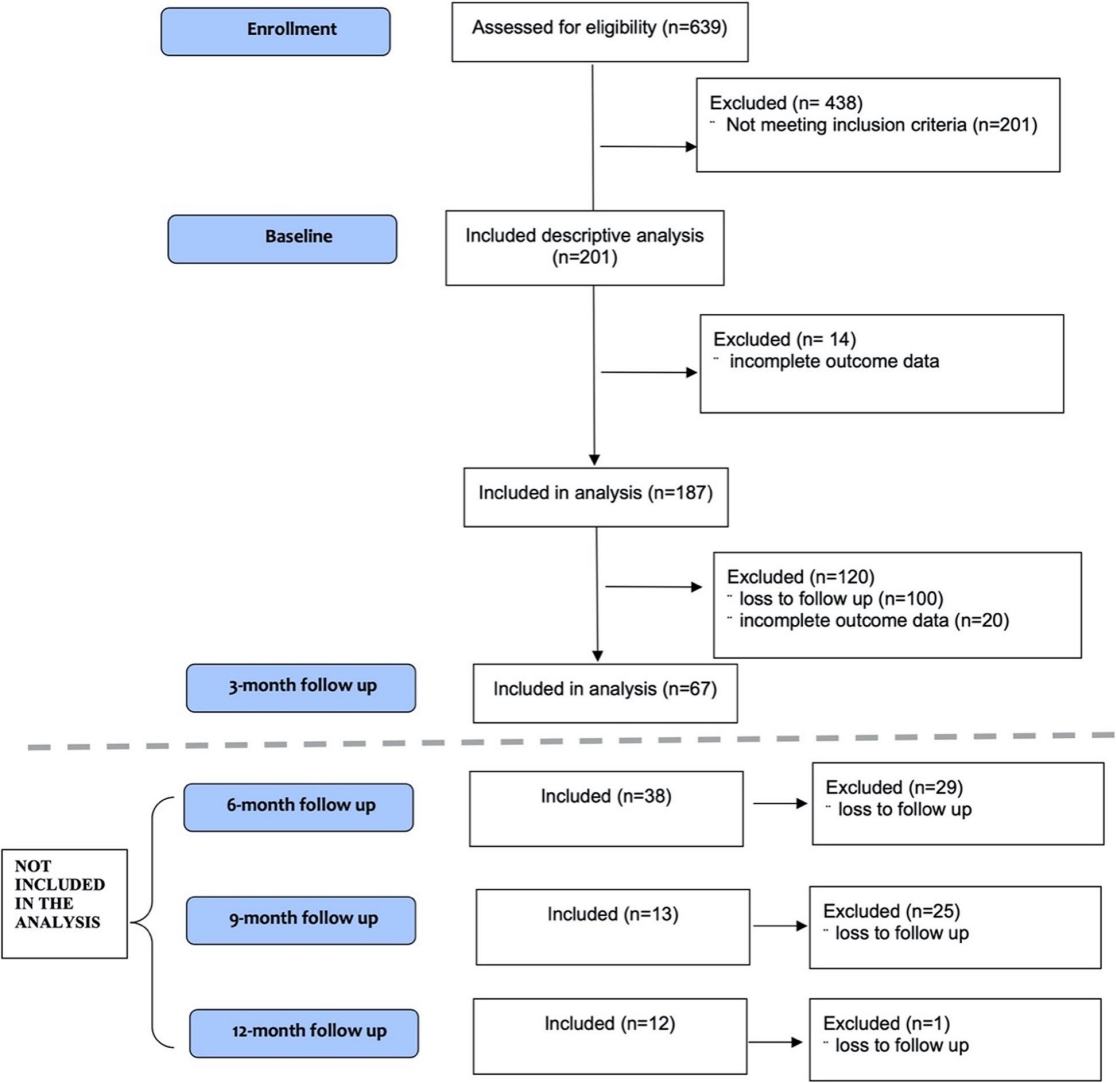
CONSORT flow diagram. The flowchart of participants disposition throughout the study. *CONSORT* Consolidated Standards of Reporting Trials

Of the 639 patients authorized Spectrum Therapeutics products between October 2017 and August 2019, 201 (31.5%) were ≥ 65 years of age and 187 had complete medical cannabis use data at baseline. The mean age was 73 years (SD = 6.83), and 121 (65.0%) were females (Table 1). Of the 187 patients with data at baseline, 65 (34.8%) provided data at the 3-month follow-up (Fig. 1). Participant characteristics of those lost to follow-up did not substantially differ from baseline or with those retained at the 3-month follow-up (Table 1).

The most common symptoms for which medical cannabis was authorized were pain (*n* = 159, 85.0%) and insomnia (*n* = 9, 4.8%). All other symptoms comprised < 5% of symptoms in the cohort (Table 1).

### Physician authorization patterns

Across the 187 patients for which complete baseline data were available, CBD-dominant products were initially authorized most frequently (99, 54%), followed by balanced (76, 42%), and then THC-dominant (8, 4.4%). Similar authorization patterns were observed at the 3-month follow-up. Patients were authorized an estimated average of 2.41 (95% CI, 1.93–2.98) mg THC and 8.42 (95% CI, 7.52–9.42) mg CBD daily at baseline. These doses more than doubled for both daily mg THC (*b* = 2.36, *t* = 5.15, *p* < .001) and daily mg CBD (*b* = 2.35, *t* = 8.34, *p* < .001) at the 3-month follow-up. Table 2 presents estimated marginal means for CBD and THC dose at baseline and 3 months. At baseline, all patients were authorized at least 1 oil, and 18% were authorized a combination of oil and dried flowers. No patients were authorized only dried flowers.

**Table 2:** Estimated marginal means and 95% confidence intervals from mixed-effects models at baseline and 3-month follow-up

Characteristic	Baseline, mean (95% CI) (***n*** = 187)	3 months, mean (95% CI) (***n*** = 67)
**Dose**		
THC (mg daily)	2.41 (1.93–2.98)	6.45 (4.59–8.95)
CBD (mg daily)	8.42 (7.52–9.42)	20.19 (16.36–24.88)
**ESAS**		
Pain	5.19 (4.78–5.60)	4.56 (3.86–5.27)
Tiredness	5.44 (5.02–5.86)	4.46 (3.83–5.08)
Drowsiness	4.53 (3.89–5.18)	3.94 (3.09–4.80)
Wellbeing	5.12 (4.72–5.52)	4.72 (4.06–5.38)
**BPI**		
Worst pain	7.68 (7.37–7.99)	6.83 (6.34–7.32)
Least pain	4.15 (3.74–4.55)	3.40 (2.70–4.10)
Average pain	6.30 (5.95–6.64)	5.47 (4.88–6.06)
Pain right now	5.11 (4.71–5.51)	4.40 (3.78–5.03)
Pain severity	5.73 (5.42–6.04)	4.97 (4.48–5.47)

*CI* Confidence interval, *ESAS* Edmonton Symptom Assessment Scale, *BPI* Brief Pain Inventory

### Outcomes of medical cannabis treatment

Table 2 presents estimated marginal means at baseline and month 3 for effects outcomes. Significant reductions in ESAS-r scores were observed over time in the domains of tiredness (*b* = -1.01, *t* = − 2.63, *p* = .013) and drowsiness (*b* = − 0.83, *t* = − 2.19, *p* = .031), but not for pain (*b* = − 0.64, *t* = − 1.65, *p* = .106) or well-being (*b* = − 0.42, *t* = − 1.11, *p* = .274).

Significant reductions in BPI-SF scores over time were observed for worst pain (*b* = − 0.87, *t* = − 2.93, *p* = .010), average pain (*b* = − 0.83, *t* = − 2.87, *p* = .012), and overall pain severity (*b* = − 0.74, *t* = − 2.72, *p* = .009). There were also reductions in pain right now (*b* = − 0.72, *t* = 1.98, *p* = .052) and least pain (*b* = − 0.64, *t* = − 1.50, *p* = .141), although these latter two did not achieve statistical significance (see Table 2).

### Differences in change from baseline between CBD-dominant and balanced product profiles

**Fig. 2:**
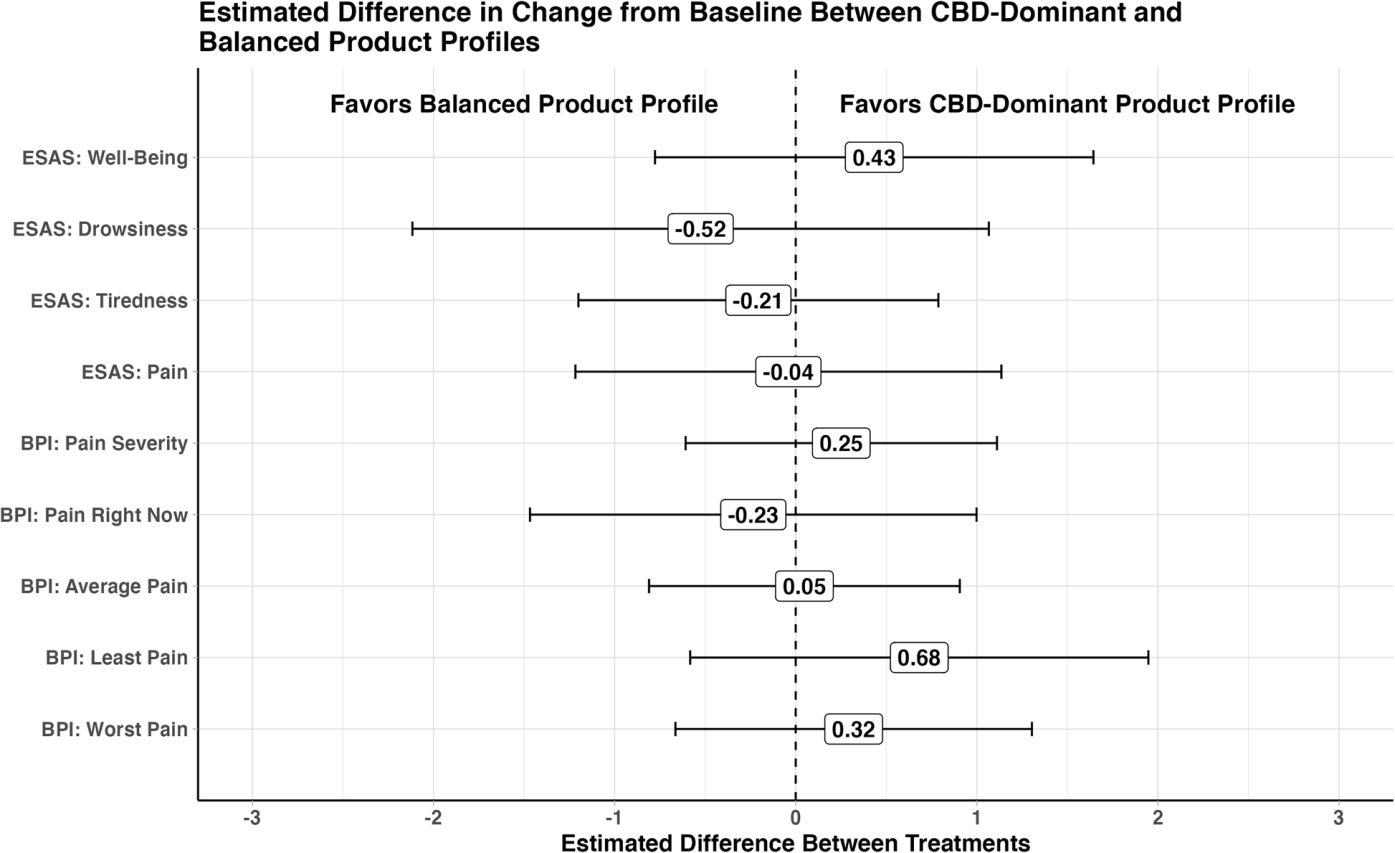
Estimated difference in change from baseline between CBD-dominant and balanced product profiles

Figure 2 depicts the estimated difference in change from baseline between baseline CBD-dominant and balanced product profile groups. As can be seen in this figure, differences between groups were modest in size and did not consistently favor one product profile over the other across outcomes (all *p*’s > .05).

### Safety

A total of 32 AEs were reported across 22 (11.7%) patients. No serious AEs were reported over the 3-month follow-up period. Table 3 shows all AEs stratified by product profile and distributed across 7 System Organ Class (SOC) categories. The most frequently reported AEs were dizziness (18.2%), nausea (9.1%), dry mouth (9.1%), and tinnitus (9.1%).

**Table 3:** Adverse events from initiation of treatment to 3-month follow-up, by MedDRA System Organ Class (SOC) and Preferred Term (PT)

		Product profile			
SOC PT	Overall *n* (%)	CBD-dominant *n* (%)	Balanced *n* (%)	THC-dominant *n* (%)	Unknown *n* (%)
**Nervous system disorders**	**10 (30.3)**	**3 (18.7)**	**5 (38.5)**	**1 (33.0)**	**1 (100.0)**
Dizziness	6 (18.2)	1 (6.2)	3 (23.1)	1 (33.0)	1 (100.0)
Headache	1 (3.0)	1 (6.2)	0 (0.0)	0 (0.0)	0 (0.0)
Disturbance in attention	2 (6.1)	1 (6.2)	1 (7.7)	0 (0.0)	0 (0.0)
Somnolence	1 (3.0)	0 (0.0)	1 (7.7)	0 (0.0)	0 (0.0)
**Gastrointestinal disorders**	**11 (33.3)**	**6 (37.5)**	**4 (30.8)**	**1 (33.0)**	**0 (0.0)**
Nausea	3 (9.1)	1 (6.2)	1 (7.7)	1 (33.0)	0 (0.0)
Dry mouth	3 (9.1)	1 (6.2)	2 (15.4)	0 (0.0)	0 (0.0)
Diarrhea	1 (3.0)	1 (6.2)	0 (0.0)	0 (0.0)	0 (0.0)
Dyspepsia	1 (3.0)	1 (6.2)	0 (0.0)	0 (0.0)	0 (0.0)
Gastrooesophageal reflux disease	1 (3.0)	1 (6.2)	0 (0.0)	0 (0.0)	0 (0.0)
Mouth ulceration	1 (3.0)	1 (6.2)	0 (0.0)	0 (0.0)	0 (0.0)
Reflux gastritis	1 (3.0)	0 (0.0)	1 (7.7)	0 (0.0)	0 (0.0)
**Psychiatric disorders**	**5 (15.2)**	**2 (12.5)**	**2 (15.4)**	**1 (33.0)**	**0 (0.0)**
Anxiety	2 (6.1)	1 (6.2)	0 (0.0)	1 (33.0)	0 (0.0)
Mood change	1 (3.0)	0 (0.0)	1 (7.7)	0 (0.0)	0 (0.0)
Aggression	1 (3.0)	0 (0.0)	1 (7.7)	0 (0.0)	0 (0.0)
Insomnia	1 (3.0)	1 (6.2)	0 (0.0)	0 (0.0)	0 (0.0)
**Ear and labyrinth disorders**	**3 (9.1)**	**2 (12.5)**	**1 (7.7)**	**0 (0.0)**	**0 (0.0)**
Tinnitus	3 (9.1)	2 (12.5)	1 (7.7)	0 (0.0)	0 (0.0)
**Respiratory, thoracic, and mediastinal disorders**	**2 (6.1)**	**2 (12.5)**	**0 (0.0)**	**0 (0.0)**	**0 (0.0)**
Cough	1 (3.0)	1 (6.2)	0 (0.0)	0 (0.0)	0 (0.0)
Dyspnea	1 (3.0)	1 (6.2)	0 (0.0)	0 (0.0)	0 (0.0)
**Metabolism and nutrition disorders**	**1 (3.0)**	**0 (0.0)**	**1 (7.7)**	**0 (0.0)**	**0 (0.0)**
Increased appetite	1 (3.0)	0 (0.0)	1 (7.7)	0 (0.0)	0 (0.0)
**Total**	**32**	**15**	**13**	**3**	**1**

*MedDRA* Medical Dictionary for Regulatory Activities, *PT* Preferred Term, *SOC* System Organ Class

There were no significant differences across product profiles

## Discussion

We completed an analysis of physician authorization patterns and self-reported symptom improvement in older adults (≥ 65 years of age) who were authorized for medical cannabis. Our findings inform the underexplored area of medical cannabis use in this population and suggest that medical cannabis is associated with therapeutic effects on pain in older adults with an acceptable safety profile, but that there is significant variability in product profile and dose consumed. Currently, there is an absence of concise clinical guidelines for physicians treating medical cannabis patients, and a perceived gap between current and desired knowledge of dosing medical cannabis (Ziemianski et al. 2015). Here, oil was the preferred product format and the most commonly authorized product at baseline was CBD-dominant, followed by balanced and only 4% of the sample was authorized a THC-dominant product. Additionally, when exploring differences across baseline product profile groups for CBD-dominant and a balanced formulation over time, neither product was favored over the other across outcomes.

We observed that the majority of patients had no previous history with cannabis use and that it appeared to be well tolerated. No serious AEs were reported, and non-serious AEs were experienced in less than 12% of the cohort. The most common AEs observed (dizziness, nausea, dry mouth, and tinnitus) were similar with the results of a longitudinal study in older adults that observed the following most common AEs: dizziness, sleepiness/fatigue, dry mouth, and psychoactive sensation (Abuhasira et al. 2018; Velayudhan et al. 2021). Moreover, these AEs were similarly observed in the general population (Wang et al. 2008). Tinnitus stood out as a unique AE in this sample and should be further investigated to determine the generalizability of this observation. Safety is a major concern for physicians when prescribing medical cannabis (Ziemianski et al. 2015); here, daily doses of up to 6.27 mg THC and 20.63 mg of CBD treatment appeared to be well tolerated.

Medical cannabis use in this sample of older adults was associated with self-reported improvement in pain over time. Significant reductions in pain symptoms, worst pain, average pain, current pain, and pain severity were observed. Additionally, reductions in tiredness were observed. These results align with several observational and randomized controlled studies that have reported improvement in pain when using medical cannabis (Whiting et al. 2015). Furthermore, pain reduction surpassed “minimal important change” levels for all BPI domains (Dworkin et al. 2008). Interestingly, there were no significant changes in well-being over time, which is inconsistent with previous studies. However, most previous studies were cross-sectional and did not measure changes over time (Bonn-Miller et al. 2014; Lum et al. 2019). Moreover, most previous studies used different instruments (i.e., the Inventory of Depression and Anxiety Scale, or a 3-point scale of overall health and well-being) to measure well-being, which may account for this discrepancy.

There are several limitations to this study. Stemming from the use of real-world data, the study lacks a control group. Additionally, patients were recruited from a single network of medical cannabis clinics in Quebec, introducing the potential for selection bias and limiting generalizability. Also, analyses of product profile differences were exploratory in nature, likely underpowered, and potentially confounded by omitted variables, and should thus be interpreted with caution. Furthermore, there was considerable attrition, a major limitation, with 67% of patients who completed the baseline assessment not completing the 3-month follow-up assessment. The degree to which the current results (among 3-month follow-up completers) can be generalized to the population of older adult medical cannabis users needs to be examined in future work with less attrition. Although the reasons for attrition were not captured, several factors can affect retention, such as financial burden, lack of effectiveness, AEs, and increased price competition from sources outside the medical cannabis program. To the extent that lack of effectiveness contributed to attrition independent of baseline symptom levels, symptom reduction estimates may be biased in an overly favorable direction. The current context of medical cannabis access in Quebec, including social stigma, high cost, and lack of universal insurance coverage can increase selection bias.

## Conclusions

These results suggest that medical cannabis was safe and well-tolerated in this sample of older adults over a 3-month period. Moreover, use of medical cannabis was associated with meaningful pain reduction, the most common primary symptom reported at baseline. However, the potential bias introduced by the high subject attrition rate means that all findings should be interpreted cautiously. It will be important to continue to examine the long-term safety and effectiveness of medical cannabis in older adults through RWE studies and to examine cause and effect relations through RCTs across a variety of health conditions.

## Data Availability

The datasets used and/or analyzed during the current study are available from the corresponding author on reasonable request.

## Abbreviations

AE: Adverse event
BPI-SF: Brief Pain Inventory Short Form
CBD: Cannabidiol
ESAS-r: Edmonton Symptom Assessment Scale-revised
MedDRA: Medical Dictional for Regulatory Activities
PT: Preferred Term
RCT: Randomized controlled trial
RWE: Real-world evidence
SOC: System Organ Class
THC: Δ-9-tetrahydrocannabinol
